# International Journal of Pediatric Endocrinology: Excellence, accessibility, expansion, and evolution

**DOI:** 10.1186/1687-9856-2011-1

**Published:** 2011-06-21

**Authors:** Scott A Rivkees

**Affiliations:** 1 Department of Pediatrics, Yale University School of Medicine, New Haven, CT USA

## 

Two years ago, the *International Journal of Pediatric Endocrinology *(*IJPE*) was launched to provide the first fully electronic, open access journal for the field in an era of evolution in media and global medical interaction. *IJPE *was launched with the incredible support of Hindawi Publishing Corporation and has quickly become a leading journal in our field. *IJPE *has rapidly evolved with the help of our readers and colleagues, to whom we are grateful for choose *IJPE *as a platform for presenting their work to the world. Since our birthday, we have had nearly 400 submissions and have published 100 reports. *IJPE *is now the official or affiliated journal of six major pediatric endocrinology societies that encompass more than 80% of the pediatric endocrinologists in the world. Having exceeded our initial goals, it is now time for IJPE to continue its evolution as the premier pediatric endocrine journal by joining forces with the Biomedical Central (BMC) family of journals.

The BMC open access journal group includes more than 100 journals, a number of which are the leading and official journals in their field. BMC journals are highly selective with numerous journals achieving lofty impact factors. BMC features contemporary electronic platforms with electronic links with other related journals, endocrine-related publications, and endocrine society home pages. This electronic system facilitates information sharing for our field at an unmatched level.

Excellence, accessibility, expansion, and evolution will be the tenets which will guide IJPE in our new partnership. To achieve excellence, we will continue to be highly selective in the reports we accept. Our goal is the dissemination of novel discoveries, be they in basic science, translational, or clinical investigation. To achieve excellence in publication standards, we will relay on our outstanding Editorial board of leading experts, who will apply high standards in report acceptance.

Accessibility will be achieved through a wonderful electronic platform that will make *IJPE *reports available in full to all with the click of the computer key at no cost. At a time when other pediatric endocrine journals charge nearly a thousand dollars or more for personal subscriptions, *IJPE *is free and will always remain free for all. Our electronic platform also ensures the near immediate dissemination of *IJPE *reports accepted for publication, allowing us to avoid lengthy publication delays associated with print in journals.

Expansion of *IJPE *will continue as we link with other pediatric endocrine societies around the globe. *IJPE *is currently affiliated with Pediatric Endocrine Society (PES), The Asia Pacific Paediatric Endocrine Society (APPES), Sociedad Chilena de Endocrinology y Diabetes (SOCHED), Indian Society for Pediatric and Adolescent Endocrinology (ISPAE), Korean Society of Pediatric Endocrinology (KSPE), and the Endocrinology Chapter of the Indonesian Pediatric Society. IJPE thus is a platform for the societies to report and disseminate important guideline reports.

It is truly our privilege and an honor to publish your work and represent your societies in *IJPE*. As such, I, BMC and our esteemed Editorial Board will continue to strive to make *IJPE your *journal for *your *individual studies and ensure that *IJPE *will be *our *journal for *our *field as our journal evolution continues.

Scott A. Rivkees, M.D.

Professor, Yale University

Editor-in-Chief, *International Journal of Pediatric Endocrinology*

